# Influence of early dose reduction of ticagrelor on clinical outcomes following percutaneous coronary intervention for complex lesions

**DOI:** 10.1038/s41598-023-42655-4

**Published:** 2023-09-19

**Authors:** Yonggu Lee, Jeong-Hun Shin, Suk Min Seo, Ik Jun Choi, Jong-Young Lee, Jun-Won Lee, Mahn-Won Park, Tae Soo Kang, Woong Gil Choi, Ki-Hyun Jeon, Hong-Seok Lim, Hyung Joon Joo, Sang Jae Rhee, Jae-Bin Seo, Myung Soo Park, Sang-Ho Park, Young-Hyo Lim

**Affiliations:** 1 Division of Cardiology, Department of Internal Medicine, Hanyang University Guri Hospital, Hanyang University College of Medicine, Guri, Republic of Korea; 2 Division of Cardiology, Department of Internal Medicine, Eunpyeong St. Mary’s Hospital, College of Medicine, The Catholic University of Korea, Seoul, Republic of Korea; 3 Division of Cardiology, Department of Internal Medicine, Incheon St. Mary’s Hospital, The Catholic University of Korea, Seoul, Korea; 4 Division of Cardiology, Department of Internal Medicine, Kangbuk Samsung Hospital, Sungkyunkwan University School of Medicine, Seoul, Republic of Korea; 5 Division of Cardiology, Department of Internal Medicine, Yonsei University Wonju College of Medicine, Wonju, Republic of Korea; 6 Division of Cardiology, Daejeon St. Mary’s Hospital, College of Medicine, The Catholic University of Korea, Daejeon, Republic of Korea; 7 Division of Cardiology, Dankook University Hospital, Dankook University College of Medicine, Cheonan, Republic of Korea; 8 Department of Internal Medicine, College of Medicine, Chungbuk National University Hospital, Chungbuk National University, Cheongju, 28644 Republic of Korea; 9 Division of Cardiology, Department of Internal Medicine, Seoul National University Bundang Hospital, Seongnam, Republic of Korea; 10 Department of Cardiology, Ajou University School of Medicine, Suwon, Republic of Korea; 11 Division of Cardiology, Department of Internal Medicine, Korea University Anam Hospital, Seoul, Republic of Korea; 12 Department of Cardiovascular Medicine, Regional Cardiocerebrovascular Center, Wonkwang University Hospital, Iksan, Republic of Korea; 13 Division of Cardiology, Department of Internal Medicine, Boramae Medical Center, Seoul National University College of Medicine, Seoul, Republic of Korea; 14 Division of Cardiology, Dongtan Sacred Heart Hospital, 7, Keunjaebong-gil, Hwaseong-si, Gyeonggi-do 18450 Republic of Korea; 15 Division of Cardiology, Department of Internal Medicine, Soonchunhyang University Cheonan Hospital, 31, Suncheonhyang 6-gil, Dongnam-gu, Cheonan, 31151 Republic of Korea; 16 Division of Cardiology, Department of Internal Medicine, Hanyang University College of Medicine, 222 Wangsimni-ro, Sungdong-gu, Seoul, 04763 Republic of Korea

**Keywords:** Cardiology, Interventional cardiology

## Abstract

Ticagrelor-based dual antiplatelet therapy (DAPT) provides potent antiplatelet inhibition but may increase the bleeding risk in Asian populations. We investigated the influence of early ticagrelor dose reduction (120 mg) on clinical outcomes in Korean patients undergoing percutaneous coronary intervention (PCI). A multicenter prospective clinical cohort study was conducted with patients who received standard-dose ticagrelor-based DAPT (180 mg) after PCI for complex lesions. Major adverse cardiovascular event (MACE: a composite of cardiovascular death, myocardial infarction, stroke, and repeat revascularization), bleeding, and net adverse clinical events (NACE: a composite of MACE and bleeding) were assessed. Among the 772 patients on standard-dose ticagrelor-based DAPT, 115 (14.8%) switched to low-dose ticagrelor-based DAPT (120 mg) within 6 months. Common reasons for the regimen changes were switching as planned (38.8%), dyspnea (25.5%), and bleeding (23.6%). A multivariable Cox proportional hazard model (CPH) showed that the risks of MACE, bleeding, and NACE were not different between the low-dose and standard-dose groups throughout the entire follow-up period and the period beyond 6 months post-PCI. Time-varying multivariable CPH models of the ticagrelor dose reduction yielded similar results. A reduction of the ticagrelor dose within 6 months after PCI is feasible and safe even in patients with complex lesions harboring a high ischemic event risk.

## Introduction

Dual antiplatelet agent therapy (DAPT) is the cornerstone of the contemporary technology for percutaneous coronary intervention (PCI). Ticagrelor is a potent P2Y12 receptor inhibitor with a binding affinity stronger than that of clopidogrel. In a landmark trial, ticagrelor-based DAPT reduced ischemic events without increasing bleeding tendency compared to clopidogrel-based DAPT in patients with acute coronary syndrome (ACS)^1^. Consequently, the current guidelines recommend potent P2Y12 inhibitors, including ticagrelor, as preferable to clopidogrel in patients with ACS undergoing PCI. However, other studies have shown that the use of ticagrelor for DAPT post-PCI was associated with a higher risk of bleeding events, particularly in Asian populations^2,3^. Given the low ischemic risk and high bleeding tendency in Asians, low-dose ticagrelor-based DAPT (ticagrelor at 120 mg daily) may provide better net clinical benefits of ischemic and bleeding events than standard-dose ticagrelor-based DAPT (ticagrelor at 180 mg daily) in Asian patients. A human study on pharmacodynamics also showed that low-dose ticagrelor had a similar efficacy for platelet inhibition compared to standard-dose ticagrelor^4^. In contrast, strong platelet inhibition is considered more effective in preventing ischemic events in patients with complex coronary lesions that, in general, harbor a higher ischemic risk^5^. In this study, we investigated the influence of the early reduction of ticagrelor on the clinical outcomes of patients after PCI for complex coronary lesions using a multicenter prospective registry.

## Methods

### Patients and data collection

From March 2019 to March 2020, 30 PCI centers in South Korea participated in the Xience **R**egistry **I**n **C**omplex Lesion of Acute Coronary Syndrome Patients wit**H** Ticagrelor (RICH; ClinicalTrials.gov identifier: NCT05746416), a multicenter, nonrandomized, prospective observational registry study to investigate the clinical outcomes of patients with ACS after PCI for complex coronary lesions. Patients 19 years of age or older with acute coronary syndrome (ACS) undergoing PCI for complex coronary lesions using everolimus-eluting stents (Xience®, Abbot Corp, Chicago, Illinois, US) and prescribed standard-dose ticagrelor-based DAPT were enrolled in the registry. Enrollment was determined after a patient had undergone PCI and before the patient was discharged from the PCI center. Patients who had undergone PCI using drug-eluting stents (DES) other than everolimus-eluting stents, and those with conditions requiring long-term oral anticoagulant therapy, a life expectancy < 1 year, or presenting with cardiogenic shock were excluded from the registry. Written informed consent was obtained from all patients before they were enrolled. The study protocols and procedures adhered to the Declaration of Helsinki. The Institutional Review Board of Hanyang University Seoul Hospital reviewed and approved the study protocols and monitored whether the center complied with the study protocols (IRB No: HYUH 2018-08-026-005).

### Procedures

All patients were administered 300 mg aspirin and 180 mg ticagrelor orally before they underwent index PCI and were prescribed standard-dose ticagrelor-based DAPT from the day after index PCI and planned to maintain it until the 3-month follow-up visit which actually varied from 35 to 180 days. Patients who discontinued standard-dose ticagrelor-based DAPT before 1 month were excluded. The start of low-dose ticagrelor-based DAPT was decided based on each attending physician’s preference.

The lesions requiring PCI were decided either angiographically or using functional studies by the attending interventional cardiologists. Successful PCI was defined as a residual stenosis < 30% with Thrombolysis in Myocardial Infarction grade 3 flow after PCI and the absence of death by MI and reintervention for the index coronary lesions during the admission period.

### Clinical events and definitions

Standard definitions of cardiovascular events were used for all clinical events^6^. Myocardial infarction (MI) was defined using the 4th universal definition of MI as previously described^7^. Repeat revascularization (RR) was defined as a new PCI for the target vessels or de novo coronary lesions. All-cause death was defined as death from any cause. Cardiovascular death was defined as death from MI, stent thrombosis, or ischemic stroke. A major adverse cardiac and cerebrovascular event (MACE) was defined as a composite of all-cause death, nonfatal MI, RR, stent thrombosis and ischemic stroke. Stent thrombosis was defined as a composite of definite, probable and possible stent thrombosis^6^. A bleeding event was defined as a bleeding event equivalent to Bleeding Academic Research Consortium (BARC) classification 2 or higher^8^. A net adverse clinical event (NACE) was defined as a composite of MACEs and bleeding events. Clinical follow-up started when a patient was discharged from the hospital after index PCI and ended when the patient experienced any clinical event or reached the end of the follow-up. The follow-up visits were scheduled at 1, 3 and 6 months and 1 and 2 years after discharge, but their timing could be adjusted based on clinical circumstances, ranging from days to weeks.

Standard definitions were used to classify coronary lesions^6^. Bifurcation was defined as the bifurcation of the major epicardial arteries, including the left main coronary artery, left anterior descending artery, left circumflex artery and right coronary artery, with a narrowing on the side branch. Chronic total occlusion (CTO) was defined as a nonthrombotic total occlusion lesion with collateral blood flow or a lesion with a duration of occlusion ≥ 3 months, and severe calcification was defined as grade ≥ 3 in Yamanaka’s method^9^. The risk for thrombotic and bleeding events after PCI was estimated using the DAPT scores^10^ and PARIS (Patterns of Non-adherence to Anti-Platelet Patients) scores^11^. A complex lesion was defined as a type B2 or C lesion in the American College of Cardiology (ACC)/American Heart Association (AHA) lesion classification as previously described^12,13^. A very complex lesion was defined as a composite of CTO, bifurcation, a number of stented coronary arteries ≥ 2, a number of coronary arteries narrowed ≥ 3, left main coronary artery stenosis, a number of stents used ≥ 3 and severe calcification.

### Statistical analysis

Patients were divided into 2 groups as follows: the standard-dose group (ticagrelor 180 mg) and the low-dose group (ticagrelor 120 mg) according to the use of low-dose ticagrelor-based DAPT within 6 months after index PCI. Patients who switched to DAPT other than ticagrelor-based DAPT (nonticagrelor-based DAPT) within the 6-month follow-up were included in the descriptive analyses but not included in the comparative analyses between the ticagrelor-based DAPT groups. Continuous variables were compared using a Student’s t test and categorical variables were compared using a chi-square test. The Mann‒Whitney U test was used for continuous variables with a skewed distribution, and Fisher’s exact test was used for categorical variables with expected values < 5 in any cells in the contingency table.

Most variables harbored missing values ≤ 1% except the low density lipoprotein (LDL) cholesterol level which had missing values of 20.2% (Fig. S1). We performed multiple imputations using a bootstrap expectation–maximization algorithm. Five possible imputed datasets were created and the average value of the 5 imputed values was adopted as the missing values for continuous variables. The most frequent value of the 5 imputed values was adopted for categorical variables. The imputation quality for the imputed variables is presented in Fig. S2.

The cumulative incidences of MACEs, bleeding events and NACEs were estimated using the Kaplan‒Meier survival analysis and compared using a log-rank test. Cox proportional hazard (CPH) models were used to identify the association of early ticagrelor dose reduction with clinical events. The proportional hazard assumption was validated in all models using the Schoenfeld residuals test. Multivariable CPH models included covariates known to influence clinical outcomes after PCI or decisions for antiplatelet regimen changes, including age, sex, body mass index, current smoking, diabetes, hypertension, kidney function, LDL cholesterol, prior PCI, clinical diagnosis, atrial fibrillation, use of statins, angiotensin blockers, beta blockers and proton-pump inhibitors (PPI), and lesion characteristics including the number of narrowed coronary arteries, total stent lengths, average stent diameters, the presence of the bifurcation lesion with side branch narrowing, chronic total occlusion, and moderate to severe calcification. The multivariable models were reduced using a backward variable selection procedure (Criterion, p > 0.05) to minimize the multicollinearity among variables and overfitting biases. The early reduction of the ticagrelor dose was set to stay in the final model during the variable selection procedure, to evaluate its adjusted association strength with clinical outcomes.

For robust adjustment of the baseline differences between the low-dose and standard-dose groups, inverse probability treatment weighting (IPTW) was applied to the dataset. A logistic model was used to conduct IPTW, and the covariates included in the multivariable CPH models were used as the denominators. Survival analyses and the CPH models were conducted using the weighted cohort. The quality of IPTW was evaluated using comparisons of the standardized mean differences (SMDs) in each variable before and after IPTW. A covariate with an SMD < 0.1 was considered well balanced.

Because the reduction of ticagrelor continuously occurred over time during the follow-up period, we performed survival analyses and produced CPH models with low-dose ticagrelor-based DAPT as a time-varying covariate to evaluate the influence of ticagrelor dose reduction on each clinical outcome throughout the follow-up period. Survival analyses and time-varying CPH regressions using a time-varying covariates were performed as previously described by Zhang et al.^14^, and more detailed methods for these analyses are described in Data S1. To evaluate whether the degrees of lesion complexity influence the results, we performed a sensitivity analysis for the univariable and multivariable CPH models in a subgroup with the very complex coronary lesions defined above.

All statistical analyses and visualizations were performed using statistical software R-4.2.2 (R Core Team, R Foundation for Statistical Computing, Vienna, Austria) in the RStudio environment (Rstudio Team, BPC, Boston, MA, US), and the “*Amelia*”, “*DataExplorer*”, “*tableone*”, “*rms*”, “*descr*”, “*survival*”, “stringr”, “*ipw*”, and “*survey*” packages were used. Statistical powers of the univariable CPH models were calculated using the “*powerCT()*” function in the “*powerSurvEpi*” package according to the Freedman method^15^. A p value < 0.05 was considered significant.

## Results

**Figure 1:**
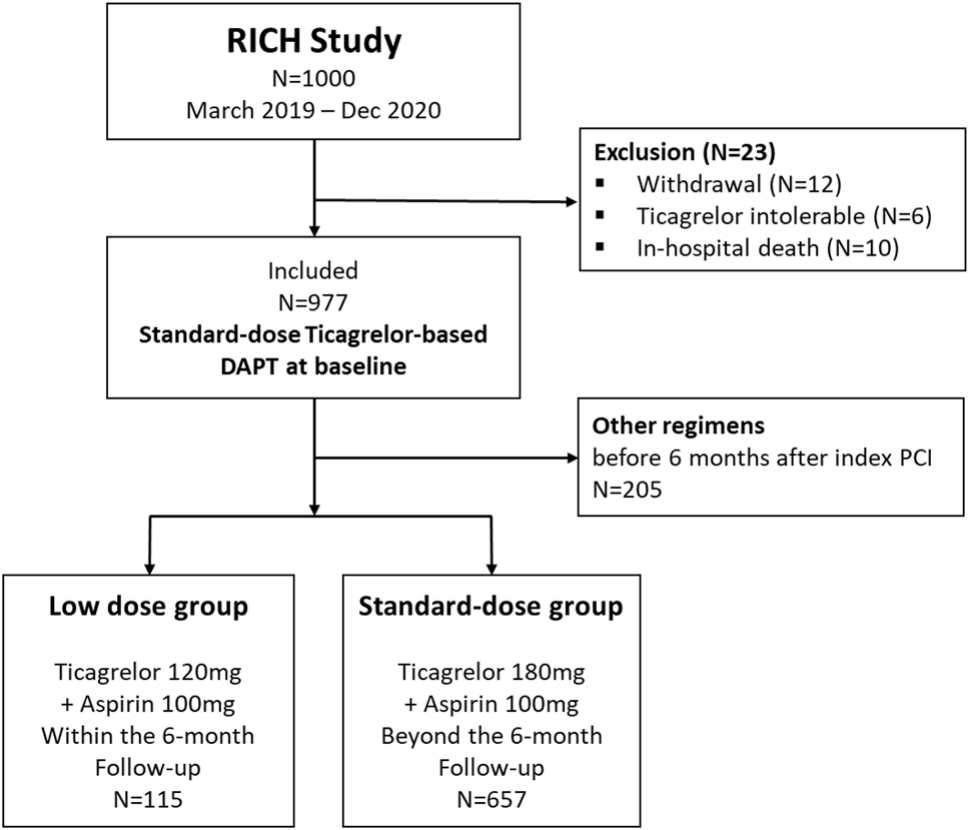
Schematic description of the patient selection process. Among the 977 patients included in the study, 208 switched their DAPT regimens to those other than ticagrelor-based DAPT before 6 months of follow-up, 115 changed to low-dose ticagrelor-based DAPT and 657 remained on standard-dose ticagrelor-based DAPT at 6 months of follow-up. The median duration of standard-dose ticagrelor use was 179 (IQR, 99–198) days in the low-dose group and 371 (IQR, 347–404) days in the standard-dose group (p < 0.001).

**Figure 2:**
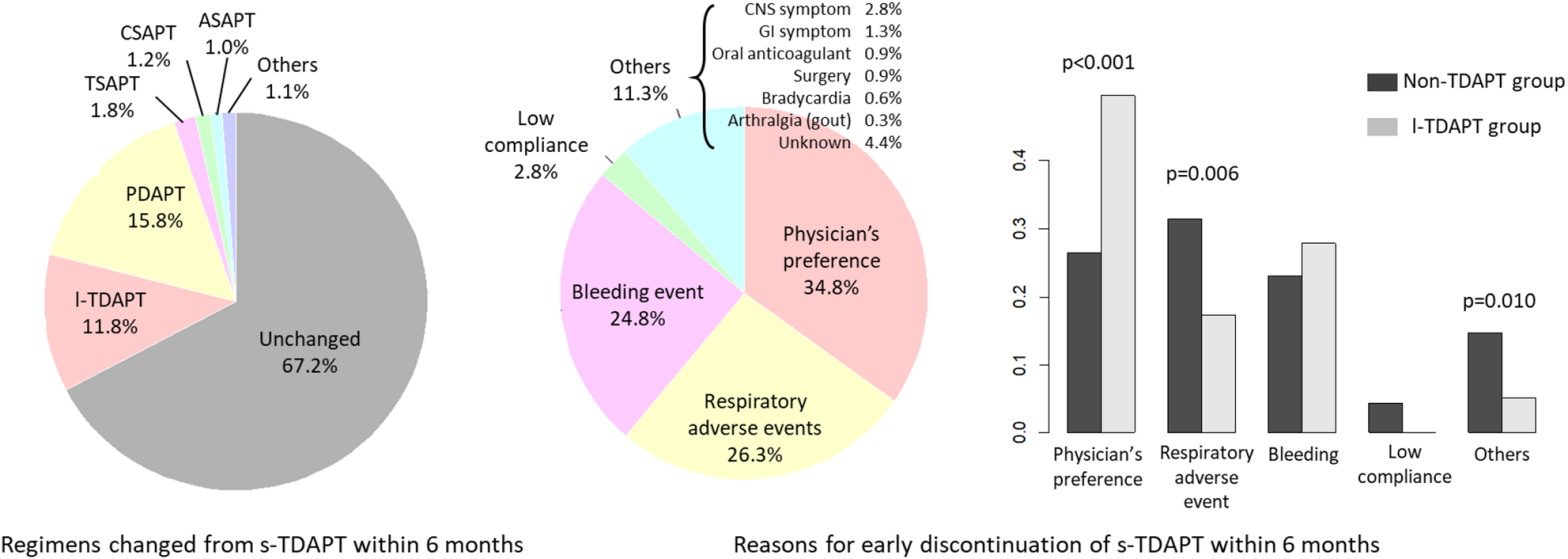
DAPT regimen change patterns within 6 months. Among the 977 patients included in the study, 32.8% switched to regimens other than standard-dose ticagrelor-based DAPT, and the 2 most common regimens to change were prasugrel-based DAPT and low-dose ticargrelor-based DAPT. The 3 most common reasons for the regimen changes were physician preference, respiratory adverse events and bleeding. Physician preference was more frequent in the ticagrelor-based DAPT group, while respiratory adverse events were more frequent in the nonticagrelor-based DAPT group. SAPT, single antiplatelet agent therapy; DAPT, dual antiplatelet therapy.

A total of 977 patients were included and followed throughout this study. Among them, 772 patients continued ticagrelor-based DAPT, and 205 patients changed to nonticagrelor-based DAPT at 6 months after index PCI (Fig. 1). The antiplatelet therapy regimen most frequently used after the regimen change was prasugrel-based DAPT (154 patients; 15.8%), and single antiplatelet therapies (SAPT) were used in 4.1% of the patients (Fig. 2) at the 6-month follow-up. Frequent reasons for early discontinuation of standard-dose ticagrelor-based DAPT were the physician’s decision (34.8%), respiratory adverse events (26.3%) including dyspnea and cough, and bleeding events (24.8%). Among those who discontinued standard-dose ticagrelor-based DAPT, the attending physician’s preference was more frequent, and respiratory side effects were less frequent in patients who switched to low-dose ticagrelor-based DAPT than in those who switched to nonticagrelor-based DAPT. The actual continuation rate of standard-dose ticagrelor-based DAPT was 76.6% of patients at 6 months, 49.5% of patients at 1 year, and 12.7% of patients at 2 years. A total of 33.7% of patients discontinued standard-dose ticagrelor-based DAPT between 9 and 15 months after index PCI (Fig. S3). After discontinuation of any ticagrelor-based DAPTs, prasugrel-based DAPT was the most preferred antiplatelet regimen. Aspirin-based SAPT was more frequently prescribed in the standard-dose group, whereas ticagrelor-based SAPT was more frequent in the low-dose group (Fig. S4).

Among those who continued ticagrelor-based DAPT at the 6-month follow-up, 657 patients (85.1%) continued standard-dose ticagrelor (standard-dose group), and 115 patients (14.9%) switched to low-dose ticagrelor DAPT (low-dose group). The median follow-up duration was 390 days (interquartile range [IQR], 359–706 days; 1030.5 person-years). The median duration of standard-dose ticagrelor-based DAPT was 371 (IQR, 347–404) days in the standard-dose group, and 179 (IQR, 99–198) days in the low-dose group. The median duration of any dose of ticagrelor-based DAPT was not different between the two groups (372 [IQR 348–407] vs. 370 [IQR, 332–400], p = 0.295).

The baseline characteristics of the patients in the standard-dose and low-dose groups are summarized in Table 1 (Table S1, including the nonticagrelor-based DAPT group). Males and the use of beta blockers, angiotensin blockers, and PPIs were more frequent in the low-dose group than in the standard-dose group. The other variables, including mean age, prevalence of comorbidities, clinical diagnosis, kidney function, HbA1c, lipid profiles, and statin use were not different between the two groups. The DAPT scores and PARIS scores for estimating the risks of thrombotic and bleeding events were also comparable between the two groups. Angiographic characteristics at index PCI are described in Table 2. Elective PCI was less frequent in the low-dose group. The number of lesions stented and the frequency of complete revascularization were greater in the low-dose group, whereas the average stent diameter and contrast media amount were greater in the standard-dose group.

**Table 1:** Baseline clinical characteristics of patients.

	Standard-dose	Low-dose	p-value	SMD
	N = 657	N = 115		
Age (year)	60.3 ± 11.6	61.3 ± 10.5	0.385	0.091
Age ≥ 60 year	330 (50.2)	65 (56.5)	0.252	0.019
Male sex	557 (84.8)	88 (76.5)	0.039	0.210
BMI (kg/m^2^)	25.0 ± 3.6	24.6 ± 3.1	0.198	0.136
Smoking			0.218	0.170
Never	236 (35.9)	40 (34.8)		
Ex-	116 (17.7)	28 (24.3)		
Current	305 (46.4)	47 (40.9)		
Comorbidities				
Hypertension	317 (48.2)	57 (49.6)	0.873	0.026
Diabetes	185 (28.2)	26 (22.6)	0.263	0.128
Atrial fibrillation	18 ( 2.7)	2 (1.7)	0.760	0.068
CKD (eGFR ≤ 60 mL/min/1.73m^2^)	80 (12.2)	11 (9.6)	0.519	0.084
ESRD	4 (0.6)	2 (1.7)	0.485	0.105
Prior MI	18 (2.7)	1 (0.9)	0.385	0.141
Prior PCI	40 (6.1)	7 (6.1)	1.000	< 0.001
Prior PVD	4 (0.6)	1 (0.9)	1.000	0.030
HF	8 (1.2)	0 (0.0)	0.490	0.157
Clinical diagnosis			0.783	0.103
Stable angina	23 (3.5)	5 (4.3)		
Unstable angina	170 (25.9)	27 (23.5)		
NSTEMI	180 (27.4)	36 (31.3)		
STEMI	284 (43.2)	47 (40.9)		
Laboratory tests				
eGFR (mL/min/1.73m^2^)	84.3 ± 21.0	85.9 ± 20.8	0.554	0.062
HbA1c (%)	6.7 ± 1.4	6.6 ± 1.2	0.538	0.066
Total cholesterol (mg/dL)	183.3 ± 47.7	186.0 ± 44.7	0.568	0.059
LDL cholesterol (mg/dL)	113.6 ± 40.8	111.4 ± 36.2	0.584	0.058
Triglyceride (mg/dL)	168.7 ± 123.5	154.5 ± 82.4	0.235	0.135
HDL cholesterol (mg/dL)	44.2 ± 10.1	44.8 ± 10.4	0.550	0.060
Medications				
Statin	631 (96.0)	112 (97.4)	0.663	0.076
Beta blocker	387 (58.9)	83 (72.2)	0.010	0.282
ACEI/ARB	372 (56.6)	78 (67.8)	0.032	0.233
PPI	316 (48.1)	74 (64.3)	0.002	0.332
PARIS bleeding score	4.1 ± 2.2	4.0 ± 1.8	0.701	0.036
PARIS coronary thrombotic event score	2.8 ± 1.2	2.7 ± 1.3	0.263	0.116
DAPT score	1.3 ± 1.3	1.3 ± 1.3	0.937	0.008
Discontinuation of standard-dose ticagrelor-based DAPT	403 (61.3)	115 (100)	< 0.001	1.123
Duration for standard-dose ticagrelor-based DAPT	371 [347, 404]	179 [99, 198]	< 0.001	1.973
Discontinuation of ticagrelor-based DAPT^a^	393 (59.8)	90 (78.3)	< 0.001	0.407
Duration for ticagrelor-based DAPT^a^	372 [348, 407]	370 [332, 400]	0.295	0.193

Data are presented as the mean ± SD or N (%).

Data with a skewed distribution are presented as the median value [Interquartile range].

DAPT, dual antiplatelet therapy; BMI, body mass index; CKD, chronic kidney disease; eGFR, estimated glomerular filtration rate; NSTEMI, non-ST segment elevation myocardial infarction; STEMI, ST segment elevation myocardial infarction; ARB, angiotensin receptor blocker; ACEI, angiotensin converting enzyme inhibitor; PPI, proton-pump inhibitor.

^a^Discontinuation of any type of ticagrelor-based DAPT (either standard-dose or low-dose) and the duration of time for which any type of ticagrelor-based DAPT was used.

**Table 2:** Baseline angiographic/procedural characteristics of patients.

	Standard-dose	Low-dose	p-value	SMD
	N = 657	N = 115		
Angiography and lesions				
PCI situation			0.029	0.266
Elective (≥ 24 h)	327 (49.8)	45 (39.1)		
Emergent (< 90 min)	235 (35.8)	56 (48.7)		
Urgent (< 24 h)	95 (14.5)	14 (12.2)		
Disease extent^a^			0.198	0.189
1 vessel disease	294 (44.7)	60 (52.2)		
2 vessel disease	229 (34.9)	39 (33.9)		
3 vessel disease	134 (20.4)	16 (13.9)		
LMCA	47 (7.2)	6 (5.2)	0.558	0.083
LAD	509 (77.5)	90 (78.3)	0.935	0.020
LCX	244 (37.1)	47 (40.9)	0.499	0.078
RCA	317 (48.2)	46 (40)	0.127	0.166
Instent restenosis	15 (2.3)	4 (3.5)	0.649	0.073
Chronic total occlusion	45 (6.8)	7 (6.1)	0.943	0.028
Bifurcation with side branch	61 (9.3)	13 (11.3)	0.497	0.016
Moderate to severe calcified lesion	63 (9.6)	17 (14.8)	0.129	0.159
Numbers of lesions	1.46 ± 0.74	1.78 ± 1.09	< 0.001	0.350
Total stent length (mm)	42.7 ± 27.7	46.9 ± 28.4	0.140	0.148
Average stent diameter (mm)	3.21 ± 0.42	3.09 ± 0.40	0.004	0.295
Complete revascularization	503 (76.6)	110 (95.7)	< 0.001	0.572
Procedures				
Contrast amount (mL)	184.1 ± 67.0	165.3 ± 57.6	0.005	0.300
Radial access only	431 (65.6)	83 (72.2)	0.204	0.142
Serious complications^b^	11 (1.7)	0 (0.0)	0.331	0.184
Vascular complications^c^	6 (0.9)	0 (0.0)	0.656	0.135

Data are presented as the mean ± SD or N (%).

^a^A left main lesion was considered a composite of LAD and LCX lesions.

^b^Cardiogenic shock, transient hypotension, pulmonary edema, cardiac tamponade, acute limb ischemia, renal injury (including dialysis) and temporary mechanical ventilation.

^c^Hematoma, AV fistula, pseudoaneurysm, deep vein thrombosis and infection.

PCI, percutaneous coronary intervention; LMCA, left main coronary artery disease; LAD, left anterior descending artery; LCX, left circumflex artery; RCA, right coronary artery; MACE, major adverse cardiovascular event; CV, cardiovascular.

**Figure 3:**
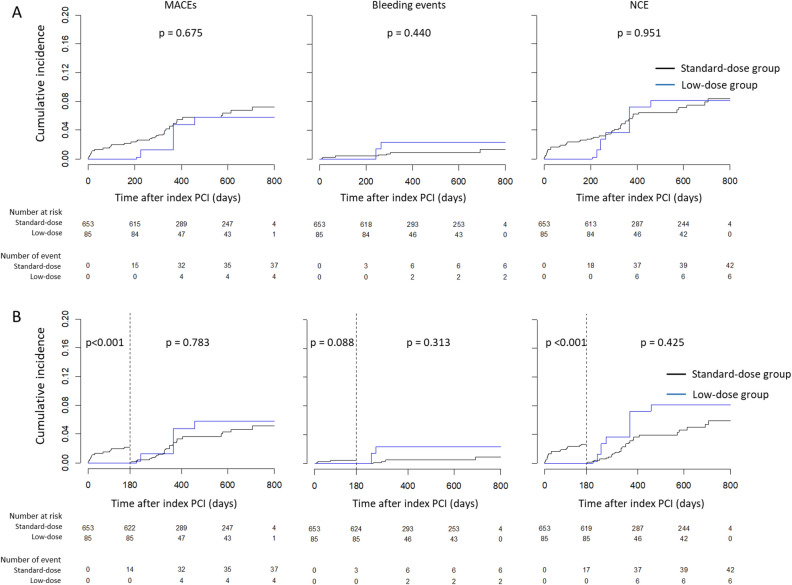
Kaplan‒Meier survival analysis for MACEs, bleeding events and NACEs in the IPTW-applied cohort. The cumulative incidences of all clinical events were not significantly different between the standard-dose and low dose groups (**A**). Landmark analyses also showed that the cumulative incidences of all clinical events were not significantly different beyond 6 months after PCI (**B**). MACE, major adverse cardiovascular event; NACE, net adverse clinical event; PCI, percutaneous coronary intervention; DAPT, dual antiplatelet agent.

The Kaplan‒Meier survival analysis showed that the cumulative incidence of MACEs, bleeding events, and NACEs were not different between the two groups (Fig. S5). The landmark analysis showed that the cumulative incidences of MACEs, bleeding events and NACEs were all comparable between the two groups in the period beyond 6 months after index PCI. After IPTW was applied, the baseline characteristics were all comparable between the groups, the median absolute standardized mean differences (SMDs) of the baseline characteristics were reduced from 0.120 to 0.067 (*p* < 0.001), and the SMDs in 33 of 43 variables were < 0.1 in the weighted cohort (Fig. S6; Table S2). Weighted Kaplan‒Meier survival analyses showed no significant differences in the cumulative incidences between the standard-dose and low-dose groups, and the landmark analyses in the period beyond 6 months showed similar results (Fig. 3).

Univariable CPH models showed that the early reduction of ticagrelor was not associated with any clinical events in either the weighted or unweighted cohort (Table 3). Multivariable CPH models also showed no significant association between the risks of clinical events and the early reduction of ticagrelor in both cohorts. Similarly, CPH models using clinical events that occurred only in the period beyond 6 months after index PCI showed no associations between the risks of any clinical events and the early reduction of ticagrelor in both cohorts.

**Table 3:** Numbers of clinical events and univariate and multivariate Cox proportional hazard models of the use of low-dose ticagrelor-based DAPT for the clinical events.

	Standard-dose	Low-dose	Unweighted cohort				Weighted cohort			
			Univariate		Multivariate^a^		Univariate		Multivariate^a^	
	N = 657	N = 115	HR (95% CI)	p	HR (95% CI)	p	HR (95% CI)	p	HR (95% CI)	p
Entire follow-up period										
MACE	40 (6.1%)	4 (3.5%)	0.53 (0.19–1.49)	0.229	0.81 (0.29–2.30)	0.695	0.78 (0.28–2.18)	0.634	0.94 (0.33–2.68)	0.904
Death	16 (2.4%)	0 (0.0%)	–	–	-	–	–	–	–	–
CV death	5 (0.8%)	0 (0.0%)	–	–	–	–	–	–	–	–
Myocardial infarction	4 (0.6%)	1 (0.9%)	–	–	–	–	–	–	–	–
Ischemic stroke	1 (0.2%)	1 (0.9%)	–	–	-	–	–	–	–	–
Repeat revascularization	22 (3.3%)	3 (2.6%)	0.70 (0.21–2.33)	0.559	0.74 (0.17–3.19)	0.689	1.01 (0.31–3.32)	0.990	0.85 (0.25–2.86)	0.789
Stent thrombosis	2 (0.3%)	0 (0.0%)	–	–	–	–	–	–	–	-
Bleeding events	7 (1.1%)	2 (1.7%)	1.59 (0.33–7.66)	0.545	1.22 (0.25–5.88)	0.804	2.33 (0.48–11.3)	0.294	2.00 (0.41–9.76)	0.389
NACE	46 (7.0%)	6 (5.2%)	0.70 (0.30–1.64)	0.415	0.90 (0.38–2.14)	0.820	1.03 (0.44–2.43)	0.940	1.18 (0.50–2.80)	0.708
Beyond 6 months										
MACE	24 (3.7%)	4 (3.5%)	0.86 (0.30–2.49)	0.785	1.01 (0.35–2.92)	0.990	1.24 (0.43–3.59)	0.694	1.33 (0.46–3.87)	0.594
Bleeding events	4 (0.6%)	2 (1.7%)	2.77 (0.51–15.1)	0.240	1.38 (0.25–7.55)	0.709	3.92 (0.71–21.6)	0.116	2.12 (0.38–11.7)	0.389
NACE	27 (4.1%)	6 (5.2%)	1.17 (0.48–2.83)	0.727	1.10 (0.46–2.67)	0.828	1.69 (0.69–4.12)	0.247	1.63 (0.67–3.97)	0.283

The multivariate model was reduced using a backward variable selection procedure (criterion p > 0.05).

MACE, major adverse cardiovascular event; CV cardiovascular; NACE, net adverse clinical event; DAPT, dual antiplatelet agent therapy.

^a^Multivariate model includes age, sex, BMI, current smoking, diabetes, hypertension, eGFR, LDL cholesterol, prior PCI, clinical diagnosis, atrial fibrillation, medications and lesion characteristics.

**Figure 4:**
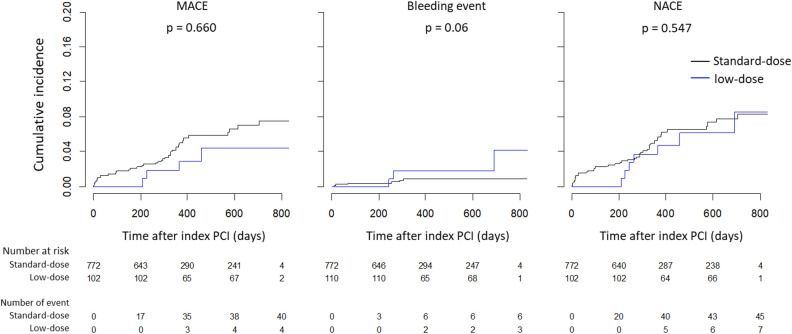
Time-varying survival curves of MACEs, bleeding events, and NACEs. A switching to low-dose ticagrelor-based DAPT occurred in 130 patients throughout the follow-up. A time-varying survival analysis showed that the cumulative incidences of MACE and NACE were not different between the standard-dose and low-dose groups, but bleeding was marginally more frequent in the low-dose group. MACE, major adverse cardiovascular event; NACE, net adverse clinical event; PCI, percutaneous coronary intervention.

Throughout the follow-up period, changes from standard-dose ticagrelor to low-dose ticagrelor occurred in 130 patients. The time-varying survival curve showed no significant differences in the cumulative incidences of MACEs and NACEs between the two groups, while bleeding events occurred marginally more frequently in the low-dose group. Multivariable time-varying CPH models also showed that the early reduction of ticagrelor was not associated with any clinical events (Fig. 4; Table 4).

**Table 4:** Multivariate time-varying Cox proportional hazard models of the use of low-dose ticagrelor-based DAPT for the clinical events.

	Univariate		Multivariate^a^	
	HR (95% CI)	p values	HR (95% CI)	p values
MACE	0.79 (0.28–2.26)	0.660	1.23 (0.43–3.54)	0.704
Bleeding events	4.88 (1.06–22.5)	0.042	3.31 (0.70–15.6)	0.130
NACE	1.29 (0.56–2.94)	0.546	1.69 (0.73–3.90)	0.219

The multivariate model was reduced using a backward variable selection procedure (criterion p > 0.05).

MACE, major adverse cardiovascular event; NACE, net clinical event.

^a^Multivariate model includes age, sex, BMI, current smoking, diabetes, hypertension, eGFR, LDL cholesterol, prior PCI, clinical diagnosis, atrial fibrillation, medications and lesion characteristics.

In the subgroup of patients with very complex lesions, patients in the low-dose group were older, more likely to be females, and less likely to be current smokers and had marginally higher prescription rates of beta-blockers and PPIs (Table S3). The number of stents, total stent lengths, and the complete revascularization rate were higher in low-dose ticagrelor users than in standard-dose ticagrelor users in the subgroup. The incidences of MACE and NACE were not significantly different between the low-dose and standard-dose groups, and multivariable CPH models showed that the early reduction of ticagrelor was not associated with an increased risk of MACEs and NACEs throughout the entire follow-up period and beyond 6 months after index PCI. The incidence of bleeding was higher in the low-dose group, but only 3 bleeding events (1 in the standard-dose group and 2 in the low-dose group) occurred beyond 6 months after index PCI in the subgroup (Table S4). The number of clinical events at 1 year after index PCI is detailed in Table S5. Univariable and multivariable CPH models indicated that risk of MACEs, bleeding events and NACEs were not significantly different between the two groups in both unweighted and weighted cohorts (Table S5).

## Discussion

We observed no differences in the risk of MACEs, bleeding events, and NACEs between the standard-dose and low-dose groups in patients undergoing PCI for complex lesions. These results were consistent with the results from patients with very complex coronary lesions and those from the robust adjustment using IPTW.

Although many studies demonstrated the efficacy and safety of various de-escalation strategies for DAPT, current clinical practice guidelines still recommend continuing DAPT based on potent P2Y12 receptor inhibitors for 1 year in patients with ACS, unless the patients have a high bleeding risk^16^. In our multicenter registry, the majority of patients with complex coronary lesions continued any form of ticagrelor-based DAPT for approximately 1 year. However, approximately 24% and 50% of patients switched their DAPT regimens at 6 months and 1 year after PCI, respectively, and > 50% of patients who switched DAPT regimens changed it before 6 months due to adverse effects, which indicates the difficulty in continuing standard-dose ticagrelor-based DAPT in patients with complex coronary lesions.

Ticagrelor has demonstrated its superiority over clopidogrel in preventing ischemic events without increasing bleeding risk in patients with ACS^1^. However, the efficacy and safety of the standard dose of ticagrelor has been questioned, especially in Asians, since Goto et al. reported that ticagrelor-based DAPT increased the risk of major and minor bleeding without decreasing ischemic events in Asian patients with ACS^2^. A more recent randomized controlled trial (RCT) showed that ticagrelor significantly increased the risk of bleeding events and numerically increased ischemic events in Korean patients with ACS^17^. Using nationwide insurance claim data, Lee et al. reported that ticagrelor-based DAPT was associated with higher risks not only of bleeding events but also of ischemic events than clopidogrel-based DAPT in Korean patients undergoing PCI^18^. Nevertheless, ticagrelor’s more rapid onset and potent inhibition of platelet function still appeals to many patients with ACS or those undergoing PCIs for complex coronary lesions, whose thrombotic risk at the acute phase is a concern. In a retrospective cohort study conducted in Chinese patients, ticagrelor-based DAPT was associated with lower MACE and MI rates than clopidogrel-based DAPT in patients undergoing PCI for bifurcation lesions^5^. Naturally, starting low-dose ticagrelor early has drawn attention as an alternative to continuing standard-dose ticagrelor until 1 year after PCI. Several studies on the pharmacokinetics of ticagrelor revealed that low-dose ticagrelor inhibited 80%–100% of P2Y12 reactivity, and there were no differences in platelet inhibition and P2Y12 reactivity between low- and standard-dose ticagrelor^4,19,20^. These results suggest that low-dose ticagrelor-based DAPT could have had a similar efficacy in preventing ischemic events as standard-dose ticagrelor-based DAPT after PCI even in patients with a high thrombotic risk. Cesaro et al. reported in a small observational study (N = 181) that only 5% of MACEs occurred without any major bleeding event in patients with high ischemic risk and previous MI who received low-dose ticagrelor-based DAPT, which suggests that low-dose ticagrelor-based DAPT is safe and effective in real-world practices for patients with high ischemic risks^21^.

Various studies have reported comparisons of P2Y12 receptor reactivity^22^, while a few have reported comparisons of clinical efficacy between standard-dose ticagrelor-based DAPT and low-dose ticagrelor-based DAPT. Our results are consistent with results from those previous studies. Bonaca et al. reported in an RCT of 21,162 patients with prior MI that prolonged ticagrelor use for 1 to 3 years after index MI significantly reduced the risk of ischemic events while increasing the risk of bleeding events to a similar degree^23^. In their study, both the risk of ischemic events and bleeding events were comparable between patients receiving 180 mg and those receiving 120 mg of ticagrelor. Wang et al. reported in a small RCT (N = 63) that there were no differences in the MACE rate and bleeding rate between the low-dose and standard-dose groups of patients with ST-segment elevation MI 6 months after PCI^24^. In their study, bleeding complications occurred in the low-dose group, although the number of events was small (4 in the low-dose group vs. 8 in the standard-dose group, p = 0.337). A retrospective cohort study showed that low-dose ticagrelor-based DAPT was as effective as standard-dose ticagrelor-based DAPT in reducing the risk of ischemic events compared to clopidogrel-based DAPT in East Asian patients undergoing PCI for CTO lesions^24^. The study also reported that the use of low-dose ticagrelor-based DAPT was significantly associated with a lower risk of bleeding events compared to standard-dose ticagrelor-based DAPT. Regarding the results from previous RCTs and observational studies, our results suggest comparable clinical efficacy between low- and standard-dose ticagrelor in patients with complex coronary lesions.

### Limitations

The current study has several limitations. First, the current study was observational, and switching the DAPT regimens was at the discretion of each physician; therefore, the regimen could have been purposefully selected by the physicians to avoid adverse events. In fact, bleeding events were more frequent in the nonticagrelor-based DAPT group than in the two ticagrelor-based DAPT groups because physicians selectively changed to nonticagrelor-based DAPT regimens in high bleeding risk patients (1.1% in the standard-dose group vs. 1.7% in the low-dose group vs. 7.3% in the nonticagrelor-based DAPT group, p < 0.001 in a log-rank test). This nonrandom regimen switching may have contributed to differences in the baseline characteristics between the two ticagrelor-based DAPT groups. To overcome baseline risk differences, we thoroughly adjusted all potential confounders in the multivariable CPH models and used IPTW to balance the baseline differences between the two groups. Second, the ischemic event rate was low (5.7% during the follow-up period and 3.8% beyond 6 months of follow-up), and the bleeding event rate was even lower; only 6 patients (1.2%) developed bleeding events beyond 6 months in the ticagrelor-based DAPT group. The nonrandom regimen switching, the short follow-up duration and the exclusion of cardiogenic shock and reintervention during index admission may have contributed to these low event rates. The fact that no clinical events occurred during the first 6 months in the low-dose ticagrelor-based DAPT group also suggests the presence of a selection bias in the decision of regimen change to low-dose ticagrelor-based DAPT. These low event rates increase the chances of beta errors. The statistical powers of the survival analyses were 0.838 for MACEs, 0.206 for bleeding events, and 0.528 for NACEs, which were generally low except for MACEs. Therefore, the nonassociations of the low-dose ticagrelor-based DAPT with bleeding events and NACEs remain inconclusive in our study, and further studies with sufficient statistical power are desired. Third, the complex coronary lesion was defined as a type B2 or C lesion from the ACC/AHA classification proposed in 1988. This classification may be obsolete for distinguishing a clinically relevant complex lesion, in contemporary PCI environments where various lesion modification techniques, intracoronary imaging and computerized tomography angiography are prevalent with the use of second generation DES^25^. However, this classification is still useful to guide PCI on site and often implemented as a lesion classifier in multicenter clinical studies. Theuerle et al. also reported more recently that the ACC/AHA classification still had prognostic value in predicting procedural successes and medium-term clinical outcomes^25^. Moreover, we conducted a sensitivity analysis in the group with very complex lesions and found consistent results with those in the entire study population. Finally, the choice of subsequent antiplatelet regimens after discontinuation of any ticagrelor-based DAPT was based on physician preferences, leading to significant differences between the groups. These disparities in the second-line antiplatelet regimens might have influenced clinical outcomes beyond 1 year after index PCI.

## Conclusions

In this multicenter prospective cohort study, we found that premature discontinuation of standard-dose ticagrelor-based DAPT frequently occurred among patients who had undergone PCI for complex coronary lesions. The early reduction of ticagrelor was not associated with increased risks of MACEs, bleeding events, and NACEs in these patients. The early reduction of ticagrelor may be a safe and feasible de-escalation strategy for ticagrelor-based DAPT in patients with high ischemic event risk, although its influence on the risk of bleeding events was not sufficiently evaluated. Because of observational natures and underpowered results of our study, a careful approach is advised for the interpretation of the results. Further studies with larger sample sizes and randomization designs are needed to elucidate the role of the early reduction of ticagrelor in de-escalation strategies for ticagrelor-based DAPT in these patients.

### Supplementary Information

Supplementary Information.

## Data Availability

The datasets generated during and/or analyzed during the current study are available from the corresponding authors on reasonable request.
