# Supplementary material for: Influence of early dose reduction of ticagrelor on clinical outcomes following percutaneous coronary intervention for complex lesions
Source: Sci Rep. 2023 Sep 19;13:15481. doi: 10.1038/s41598-023-42655-4 (PMC10509174; doi:10.1038/s41598-023-42655-4)
Supplement: Supplementary file 1 — Supplementary Information. [file 41598_2023_42655_MOESM1_ESM.docx]

**Supplementary Data**

**Supplementary Data**

Data S1. Descriptions of time-varying covariates and time-varying Cox proportional hazard models

**Supplementary Figure**

Figure S1. Frequencies of missing variables in the final dataset

Figure S2. Multiple imputation results for the major variables with missing values

Figure S3. Survival rate of standard-dose ticagrelor-based DAPT

Figure S5. Frequency of antiplatelet agent regimens followed after the discontinuation of ticagrelor-based DAPT

Figure S4. Kaplan‒Meier survival analysis for MACEs, bleeding events, and NACEs in the unweighted cohort

Figure S5. SMDs in baseline characteristics between the two groups in the weighted and unweighted cohorts.

**Supplementary Tables**

Table S1. Baseline clinical characteristics of all patients, including the nonticagrelor-based DAPT group

Table S2. Baseline clinical characteristics of patients in the IPTW-applied cohort

Table S3. Baseline clinical characteristics of patients with very complex coronary lesions

Table S4. Clinical outcomes of patients with very complex coronary lesions

Table S5. Numbers of clinical events and univariate and multivariate Cox proportional hazard models of the use of low-dose ticagrelor-based DAPT for the clinical events until 1 year

**Supplementary Data**

Data S1. Descriptions of time-varying covariates and time-varying Cox proportional hazard models

This is a brief description of the purpose of time-varying Cox proportional hazard (CPH) models and how to create a survival dataset with a time-varying covariate, perform survival analysis and produce CPH models with the time-varying covariate. We provide actual R software codes and part of the dataset that we had used to produce our results.

1. Purpose

Time-varying covariates are variables that change their values over time during the observation period, which are frequently found in longitudinal clinical study cohorts. For example, in patients with atrial fibrillation, the use of cardiac digitalis may be either beneficial or harmful for patient’s survival. The hypothesis can definitely be tested in a double-blinded randomized control trial, but in a clinical registry of patients with newly diagnosed atrial fibrillation, the start of digitalis may occur sometime after diagnosis during the observation period. Simple comparisons of clinical outcomes between patients prescribed digitalis and those not prescribed may be susceptible to bias because the use of digitalis may occur after the clinical outcomes and may also indicate some differences in the disease status at the time of start. Therefore, time-varying covariates are useful in such situations.

In the current study, the reduction of ticagrelor from the standard-dose (180mg) to the low-dose (120mg) in dual-antiplatelet agent therapy (DAPT) occurred at the discretion of each attending physician over time during the follow-up period. In some cases, it occurred even after repeat revascularization already occurred. Therefore, we performed a survival analysis and produced CPH models with low-dose ticagrelor-based DAPT as a time-varying covariate in this study.

1. How to create a survival dataset with a time-varying covariate

We created a dataset with time-varying covariate using the “*tmerge()*” function in the “*Survival*” package. The change from the standard-dose ticagrelor-based DAPT to the low-dose ticagrelor-based DAPT throughout the follow-up period was set to the time-varying exposure (the reduction of ticagrelor from the standard-dose to the low-dose), and a different survival dataset with the time-varying covariate was created for each clinical outcome.

# Time-varying covariate for major adverse cardiovascular event (MACE)

library (survival)

# gx_time: the time duration until the standard-dose was reduced to the low-dose in the ticagrelor-based DAPT. If the ticagrelor dose did not change, the value was set to “NA”.

# mace_dur: Time to the development of MACE

# mace: The development of MACE

# id: The identification variable

# dt: Original dataset containing variables including “id”, “mace_dur”, “mace”, “gx_time”, “gx” and other covariates

var<-c(“id”, “mace_dur”, “gx_time”, “mace”)

dta <- tmerge(

data1 = dt,

data2 = dt [, var],

id = id,

mace.tv = event (mace_dur, mace),

TDAPT.tv = tdc (gx_time),

Options = list (idname = “id”)

)

>dta[515:530,]

id age sex mace_dur mace tstart tstop mace.tv TDAPT.tv

515 17-1504 63 1 729 0 0 728 0 0

516 17-1497 34 1 702 0 0 24 0 0

517 17-1497 34 1 702 0 24 702 0 1

518 17-1495 49 1 735 0 0 735 0 0

519 17-1477 66 1 749 0 0 14 0 0

520 17-1477 66 1 749 0 14 749 0 1

521 17-1467 44 1 744 0 0 126 0 0

522 17-1467 44 1 744 0 126 744 0 1

523 17-1380 58 1 767 0 0 170 0 0

524 17-1380 58 1 767 0 170 767 0 1

525 17-1378 62 0 458 1 0 188 0 0

526 17-1378 62 0 458 1 188 458 1 1

527 17-1275 48 1 725 0 0 252 0 0

528 17-1275 48 1 725 0 252 725 0 1

529 17-1272 62 1 717 0 0 373 0 0

530 17-1272 62 1 717 0 373 716 0 1

# “dta”: Survival dataset with the time-varying covariate

# “tstart”: Starting time of the observation

# “tstop”: Stopping time of the observation

# “mace.tv”: MACE in the time-varying survival dataset (Outcome)

# “TDAPT.tv”: Reduction of the ticagrelor dose (Exposure)

In the time-varying survival dataset, the periods before the time-varying exposure occurred (reduction of ticagrelor) were assigned to the nonexposure group (TDAPT.tv=0) and the periods after the exposure occurred were assigned to the exposure group (TDAPT.tv=1) in patients who experienced the reduction of ticagrelor. In contrast, the entire observation period was assigned to the nonexposure group in patients who had not experienced the reduction of ticagrelor. In cases in which MACE occurred before the patients experienced the exposure, the periods until the occurrence of MACE were assigned to the nonexposure group, but the periods after the occurrence of MACE were discarded from the dataset. Subsequently, the numbers at risk in each group are different from those in the original survival dataset, and in some cases, the numbers at risk increase over time during a period of observation.

1. Survival analysis and CPH model

#Kaplan-Meier survival curves

col<-c(rgb(0,0,0,1), rgb(0,0,1,1))

xtick<-seq(0, 800, by=200)

ytick<-seq(0, 0.20, by=0.04)

fit<-survfit(Surv(tstart, tstop, mace.tv)~TDAPT.tv, data=dta)

plot(fit, fun="event", col=col, frame=F, xaxt="n", yaxt="n", ylim=c(0,0.20))

axis(1, xtick)

axis(2, ytick)

#Univariable CPH model

coxph_mace_tv<-coxph(Surv(tstart, tstop, mace.tv)~TDAPT.tv, data=dta)

summary(coxph_mace_tv)

**Supplementary Figures**


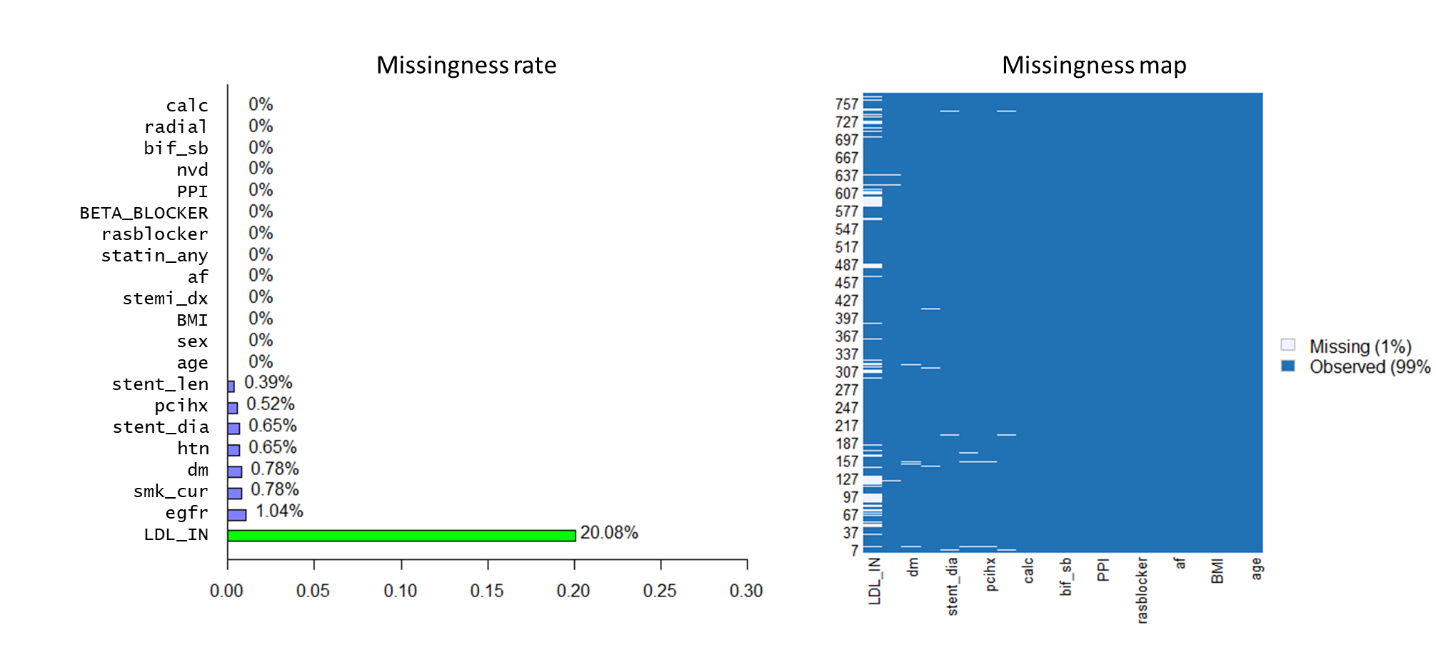


Figure S1. Frequencies of missing variables in the final dataset

Approximately 1% of the data were missed in the final dataset (N=772) for the analyses. The missing rates were <1% in most variables with missing values, but the LDL cholesterol level had missing values for 20.08% of the data.


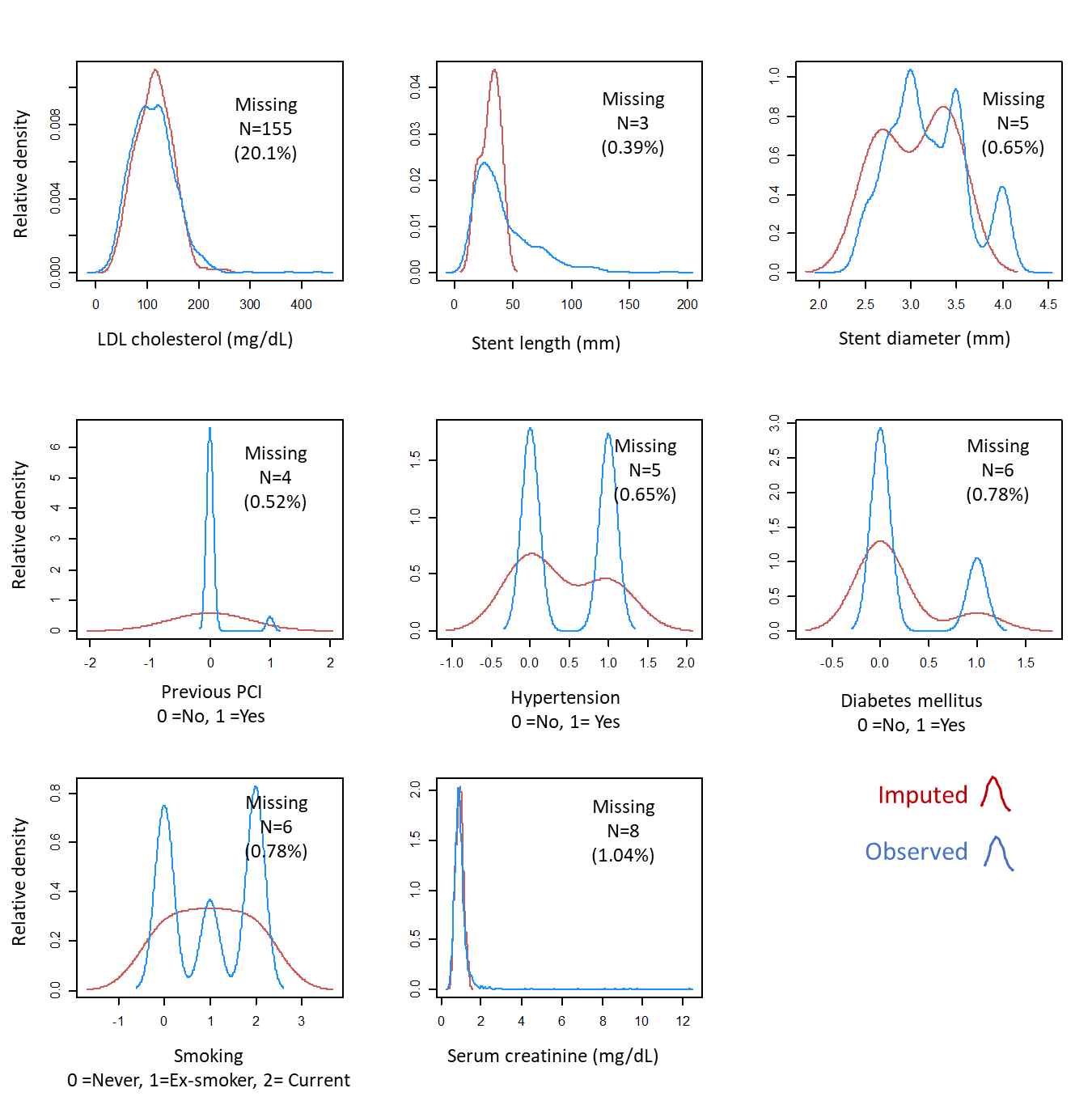


Figure S2. Multiple imputation results for the major variables with missing values

Multiple imputation was performed using a bootstrap expectation-maximization algorithm. Density plots comparing the distributions of the observed and imputed values show adequate agreements between the observed and imputed values (red plots for the imputed values and blue plots for the observed values).

N (%) indicates the number of missing values (the proportion of missing values).


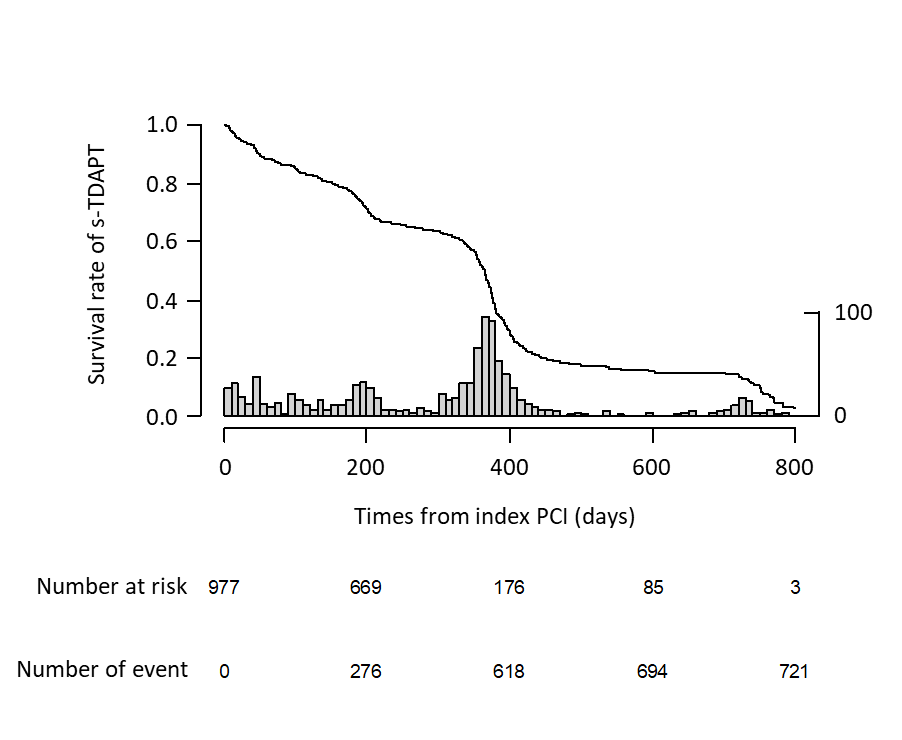


Figure S3. Survival rate of the standard-dose ticagrelor-based DAPT

The follow-up visits were scheduled at 1, 3, 6, 12, and 24 months; but actual follow-up dates varied from days to a few weeks. Therefore, the actual continuation rate of the standard-dose ticagrelor-based DAPT was plotted using a Kaplan‒Meier survival estimator. The histogram indicates the number of discontinuation of standard-dose ticagrelor-based DAPT at the time.

Standard-dose ticagrelor-based DAPT continued for 76.6% of patients at 6 months and 49.5% of patients at 1 year. More than 30% of patients discontinued the standard dose of ticagrelor-based DAPT approximately 1 year after index PCI.


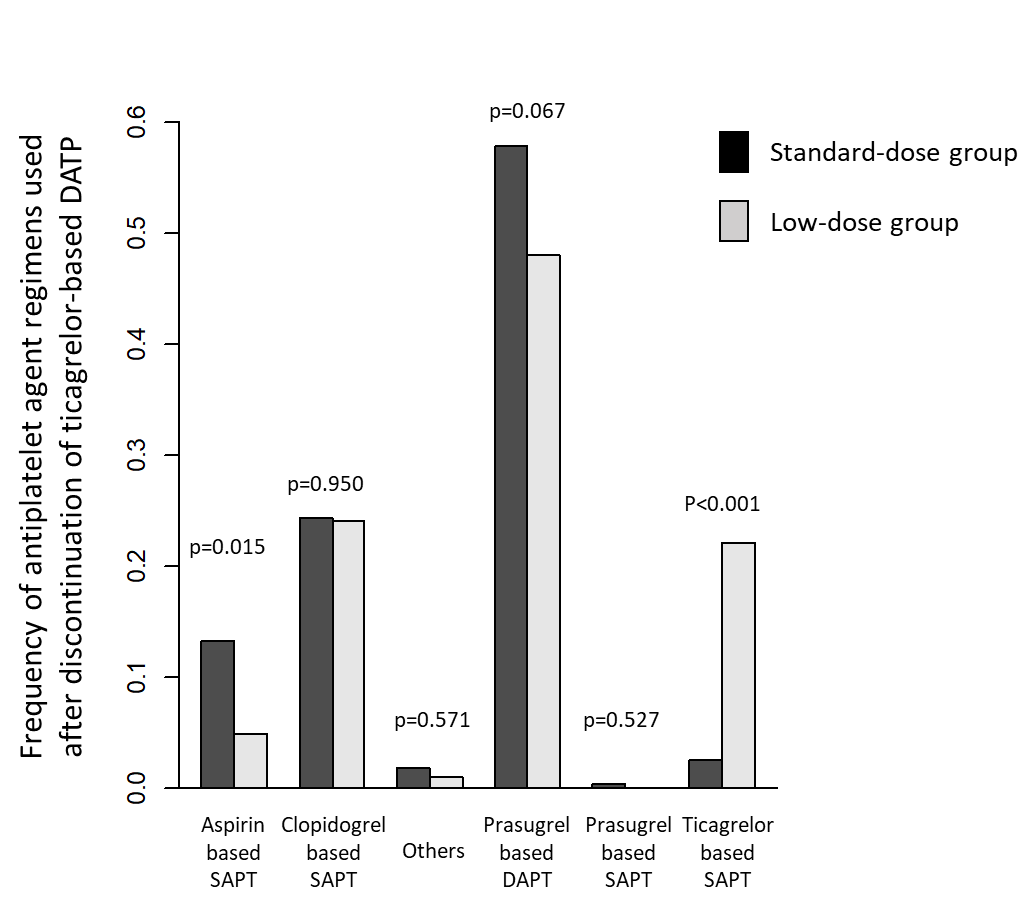


Figure S4. Frequency of antiplatelet agent regimens followed after the discontinuation of ticagrelor-based DAPT.

The antiplatelet regimens that were most frequently used was prasugrel-based DAPT, while aspirin-based SAPT was favored in the standard-dose group and ticagrelor-based SAPT was preferred in the low-dose group. Chi-square tests were used to compare the ratios of each antiplatelet regimen against all other regimens between the groups. The overall patterns of antiplatelet agent use are significantly different between the groups after the discontinuation of any ticagrelor-based DAPT (*p* <0.001 in a Chi-square test).

SAPT, single antiplatelet agent therapy; DAPT, dual antiplatelet agent therapy


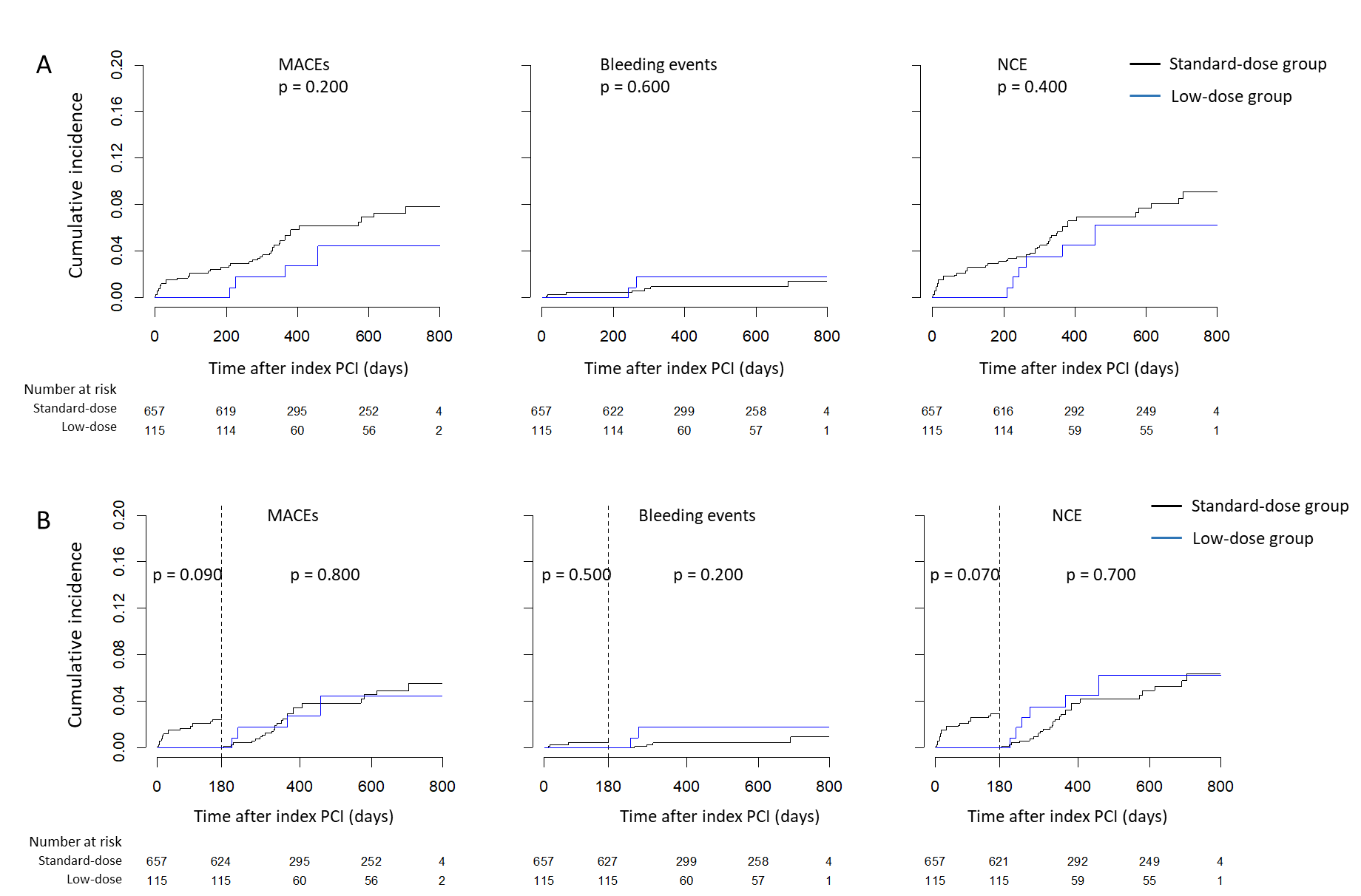


Figure S5. Kaplan‒Meier survival analysis for MACEs, bleeding events and NACEs in the unweighted cohort

Cumulative incidences of MACEs, bleeding, and NACEs were not significantly different between the standard-dose and low-dose groups (A). Landmark analyses showed that the cumulative incidences of MACEs and NACEs were marginally higher in the standard-dose group than in the low-dose group until 6 months after PCI, whereas they were not significantly different beyond 6 months after PCI (B). The cumulative incidence of bleeding events was not significantly different between groups in either period.

MACE, major adverse cardiovascular event; NACE, net adverse clinical event; PCI, percutaneous coronary intervention


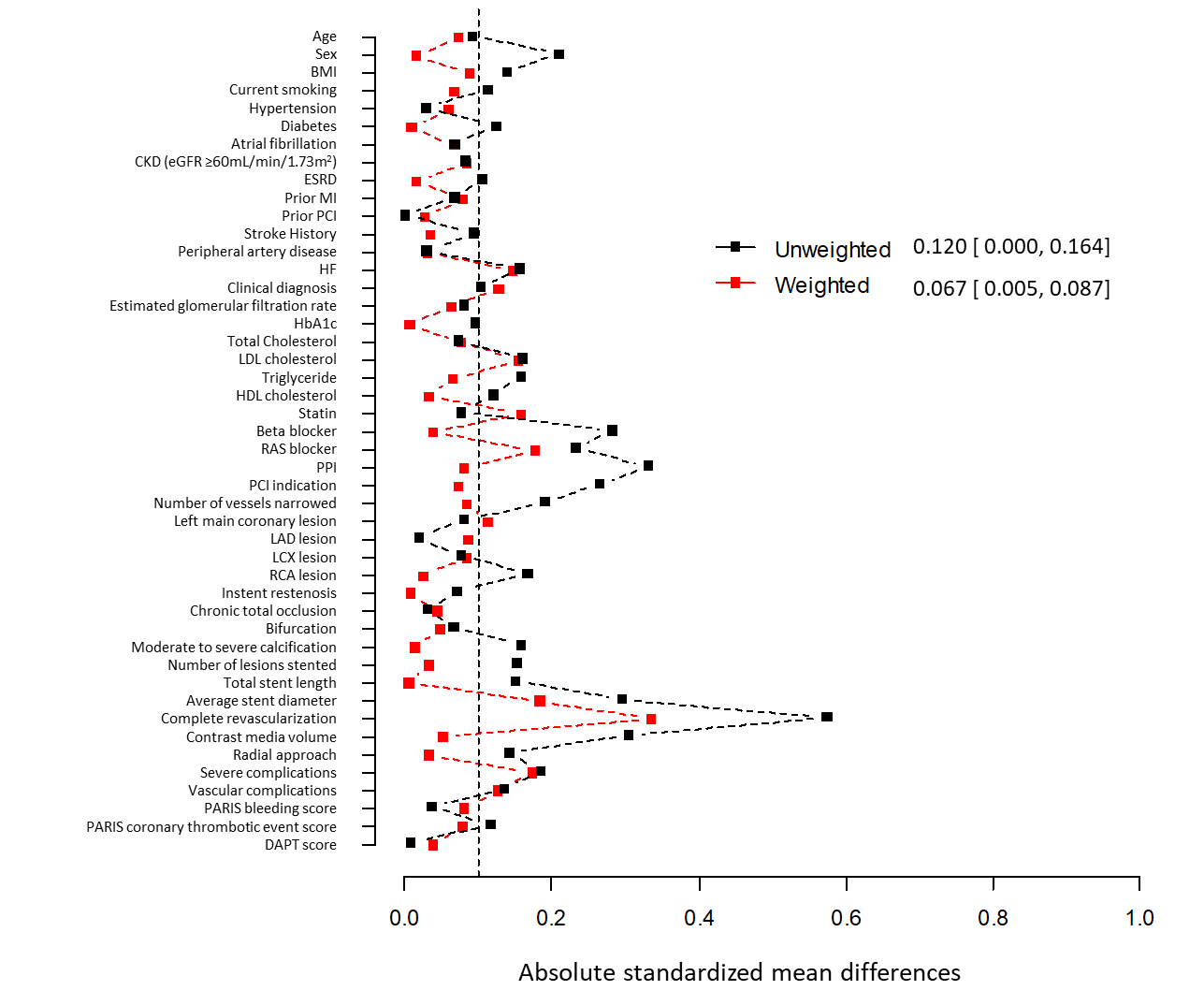


Figure S6. SMDs in baseline characteristics between the two groups in the weighted and unweighted cohorts

SMDs were significantly lower in the weighted cohort than in the unweighted cohort, and most variables’ SMDs were reduced <0.1 in the weighted cohort.

**Supplementary Tables**

| Table S1. Baseline clinical characteristics of patients in the entire cohort including the nonticagrelor-based DAPT group | | | | |
| --- | --- | --- | --- | --- |
|  | Standard-dose | low-dose | nonticagrelor |  |
|  | N=657 | N=115 | N=205 | p values |
| Clinical characteristics |  |  |  |  |
| Age (year) | 60.3±11.6 | 61.3±10.5 | 64.3±10.9 | <0.001 |
| Age ≥75 years | 330 (50.2) | 65 (56.5) | 138 (67.3) | <0.001 |
| Male sex | 557 (84.8) | 88 (76.5) | 162 (79.0) | 0.031 |
| BMI (kg/m^2^) | 25±3.6 | 24.6±3.1 | 24.8±3.0 | 0.346 |
| Smoking |  |  |  | 0.001 |
| Never | 236 (35.9) | 40 (34.8) | 101 (49.3) |  |
| Former | 116 (17.7) | 28 (24.3) | 41 (20.0) |  |
| Current | 305 (46.4) | 47 (40.9) | 63 (30.7) |  |
| Hypertension | 316 (48.1) | 57 (49.6) | 108 (52.7) | 0.517 |
| Diabetes | 184 (28.0) | 26 (22.6) | 48 (23.4) | 0.264 |
| Atrial fibrillation | 18 (2.7) | 2 (1.7) | 5 (2.4) | 0.815 |
| CKD (eGFR ≤60 mL/min/1.73m^2^) | 86 (13.1) | 12 (10.4) | 30 (14.6) | 0.565 |
| ESRD | 4 (0.6) | 2 (1.7) | 2 (1.0) | 0.445 |
| Prior MI | 18 (2.7) | 2 (1.7) | 9 (4.4) | 0.339 |
| Prior PCI | 40 (6.1) | 7 (6.1) | 17 (8.3) | 0.526 |
| Prior PVD | 4 (0.6) | 1 (0.9) | 1 (0.5) | 0.915 |
| HF | 8 (1.2) | 0 (0.0) | 2 (1.0) | 0.487 |
| Clinical diagnosis |  |  |  | 0.942 |
| Stable angina | 23 (3.5) | 5 (4.3) | 7 (3.4) |  |
| Unstable angina | 170 (25.9) | 27 (23.5) | 58 (28.3) |  |
| NSTEMI | 180 (27.4) | 36 (31.3) | 56 (27.3) |  |
| STEMI | 284 (43.2) | 47 (40.9) | 84 (41.0) |  |
| eGFR (mL/min/1.73m^2^) | 83.8±21.5 | 85.5±21.2 | 78.5±20.8 | 0.003 |
| HbA1c (%) | 6.7±1.4 | 6.5±1.2 | 6.5±1.1 | 0.346 |
| Total cholesterol (mg/dL) | 183.5±47.8 | 186.8±45 | 176±45.7 | 0.076 |
| LDL cholesterol (mg/dL) | 113.7±40.9 | 107.6±34.8 | 111.9±46 | 0.338 |
| Triglyceride (mg/dL) | 169.1±123.1 | 152.4±85.4 | 157.1±93.1 | 0.196 |
| HDL cholesterol (mg/dL) | 44.3±10.2 | 45.6±10.3 | 43.4±9.6 | 0.175 |
| Statin | 631 (96) | 112 (97.4) | 199 (97.1) | 0.658 |
| Beta blocker | 387 (58.9) | 83 (72.2) | 127 (62.0) | 0.026 |
| ACEI/ARB | 372 (56.6) | 78 (67.8) | 122 (59.5) | 0.076 |
| PPI | 316 (48.1) | 74 (64.3) | 120 (58.5) | 0.001 |
| Angiographic characteristics |  |  |  |  |
| PCI situation |  |  |  | 0.013 |
| Elective (≥24 hours) | 327 (49.8) | 45 (39.1) | 82 (40.0) |  |
| Emergent (<90 min) | 235 (35.8) | 56 (48.7) | 83 (40.5) |  |
| Urgent (<24 hours) | 95 (14.5) | 14 (12.2) | 40 (19.5) |  |
| Disease extent* |  |  |  | 0.382 |
| 1 vessel disease | 294 (44.7) | 60 (52.2) | 90 (43.9) |  |
| 2 vessel disease | 229 (34.9) | 39 (33.9) | 69 (33.7) |  |
| 3 vessel disease | 134 (20.4) | 16 (13.9) | 46 (22.4) |  |
| LMCA | 47 (7.2) | 6 (5.2) | 15 (7.3) | 0.734 |
| LAD | 509 (77.5) | 90 (78.3) | 145 (70.7) | 0.121 |
| LCX | 244 (37.1) | 47 (40.9) | 89 (43.4) | 0.246 |
| RCA | 317 (48.2) | 46 (40) | 108 (52.7) | 0.093 |
| In stent restenosis | 15 (2.3) | 4 (3.5) | 6 (2.9) | 0.704 |
| Chronic total occlusion | 45 (6.8) | 7 (6.1) | 15 (7.3) | 0.916 |
| Bifurcation | 61 (9.3) | 13 (11.3) | 22 (10.7) | 0.708 |
| Severe calcified lesions | 63 (9.6) | 17 (14.8) | 21 (10.2) | 0.240 |
| Numbers of lesions | 1.5±0.7 | 1.8±1.1 | 1.4±0.7 | <0.001 |
| Total stent length (mm) | 42.6±27.7 | 46.9±28.4 | 41.2±25.2 | 0.197 |
| Average stent diameter (mm) | 3.2±0.4 | 3.1±0.4 | 3.2±0.4 | 0.013 |
| Complete revascularization | 503 (76.6) | 110 (95.7) | 162 (79.0) | <0.001 |
| Contrast amount (mL) | 184.3±66.7 | 165.3±57.6 | 169.2±67.6 | 0.001 |
| Radial only | 431 (65.6) | 83 (72.2) | 127 (62) | 0.182 |
| Access site crossover | 259 (39.4) | 6 (5.2) | 61 (29.8) | <0.001 |
| Serious complications** | 11 (1.7) | 0 (0.0) | 3 (1.5) | 0.378 |
| Vascular complications† | 6 (0.9) | 0 (0.0) | 2 (1.0) | 0.582 |
| Clinical outcomes |  |  |  |  |
| MACE | 40 (6.1) | 4 (3.5) | 9 (4.4) | 0.398 |
| Death | 16 (2.4) | 0 (0.0) | 3 (1.5) | 0.186 |
| CV death | 5 (0.8) | 0 (0.0) | 3 (1.5) | 0.363 |
| Myocardial infarction | 4 (0.6) | 1 (0.9) | 1 (0.5) | 0.915 |
| Ischemic stroke | 1 (0.2) | 1 (0.9) | 2 (1.0) | 0.194 |
| Repeat revascularization | 22 (3.3) | 3 (2.6) | 3 (1.5) | 0.363 |
| Stent thrombosis | 2 (0.3) | 0 (0.0) | 1 (0.5) | 0.751 |
| Bleeding | 7 (1.1) | 2 (1.7) | 15 (7.3) | <0.001 |
| Composite event | 46 (7.0) | 6 (5.2) | 21 (10.2) | 0.189 |
| Data were presented as the mean ± SD or N (%). | | | | |
| *A left main lesion was considered as a composite of LAD and LCX lesions. | | | | |
| ** including cardiogenic shock, transient hypotension, pulmonary edema, cardiac tamponade, acute limb ischemia, renal injury (including dialysis) and temporary mechanical ventilation | | | | |
| † including hematoma, AV fistula, pseudoaneurysm, deep vein thrombosis and infection | | | | |
| BMI, body mass index; CKD, chronic kidney disease; eGFR, estimated glomerular filtration rate; NSTEMI, non-ST segment elevation myocardial infarction; STEMI, ST segment elevation myocardial infarction; ARB, angiotensin receptor blocker; ACEI, angiotensin converting enzyme inhibitor; PPI, proton-pump inhibitor; PCI, percutaneous coronary intervention; LMCA, left main coronary artery disease; LAD, left anterior descending artery; LCX, left circumflex artery; RCA, right coronary artery; MACE, major adverse cardiovascular event; CV, cardiovascular | | | | |

| Table S2. Baseline clinical characteristics of patients in the IPTW-applied cohort | | | | |
| --- | --- | --- | --- | --- |
|  | Standard-dose | low-dose |  |  |
|  | N=653 | N=85 | p values | SMD |
| Clinical characteristics |  |  |  |  |
| Age (years) | 60.5±11.5 | 61.3±10.2 | 0.524 | 0.073 |
| Male sex | 550 (84.3) | 72 (84.8) | 0.885 | 0.015 |
| BMI (kg/m^2^) | 25±3.5 | 24.7±3 | 0.442 | 0.088 |
| Smoking | 299 (45.8) | 36 (42.4) | 0.599 | 0.067 |
| Hypertension | 316 (48.5) | 44 (51.4) | 0.641 | 0.059 |
| Diabetes | 181 (27.7) | 23 (27.3) | 0.945 | 0.009 |
| Atrial fibrillation | 18 (2.7) | 2 (1.7) | 0.568 | 0.068 |
| CKD (eGFR ≤60 mL/min/1.73m^2^) | 85 (13.0) | 9 (10.3) | 0.529 | 0.084 |
| ESRD | 5 (0.8) | 1 (0.6) | 0.828 | 0.016 |
| Prior MI | 18 (2.7) | 4 (4.1) | 0.589 | 0.078 |
| Prior PCI | 41 (6.3) | 6 (7.0) | 0.838 | 0.027 |
| Prior CVA | 20 (3.1) | 3 (3.8) | 0.822 | 0.035 |
| Prior PVD | 4 (0.6) | 0 (0.4) | 0.683 | 0.031 |
| HF | 7 (1.1) | 0 (0.0) | 0.312 | 0.147 |
| Clinical diagnosis |  |  | 0.742 | 0.127 |
| Stable angina | 23 (3.5) | 2 (2.8) |  |  |
| Unstable angina | 172 (26.4) | 26 (30.2) |  |  |
| NSTEMI | 174 (26.7) | 25 (29.1) |  |  |
| STEMI | 283 (43.4) | 32 (37.9) |  |  |
| eGFR (mL/min/1.73m^2^) | 83.7±21.7 | 85.1±21.4 | 0.633 | 0.063 |
| HbA1c (%) | 6.7±1.4 | 6.7±1.2 | 0.955 | 0.007 |
| Total cholesterol (mg/dL) | 183±47.5 | 179.6±40.5 | 0.483 | 0.076 |
| LDL cholesterol (mg/dL) | 112.1±40.2 | 106.5±31.9 | 0.150 | 0.155 |
| Triglyceride (mg/dL) | 136 [94, 196] | 134 [100, 189] | 0.832 | 0.000 |
| HDL cholesterol (mg/dL) | 44.3±10.2 | 44.6±10.3 | 0.795 | 0.033 |
| Statin | 629 (96.3) | 84 (98.8) | 0.063 | 0.158 |
| Beta blocker | 396 (60.7) | 53 (62.6) | 0.770 | 0.039 |
| ACEI/ARB | 381 (58.3) | 57 (66.9) | 0.180 | 0.177 |
| PPI | 330 (50.6) | 46 (54.6) | 0.537 | 0.080 |
| PARIS bleeding score | 4.2±2.2 | 4.0±1.7 | 0.437 | 0.081 |
| PARIS coronary thrombotic event score | 2.8±1.2 | 2.7±1.4 | 0.596 | 0.078 |
| DAPT score | 1.3±1.3 | 1.3±1.4 | 0.778 | 0.039 |
| Angiographic characteristics |  |  |  |  |
| PCI situation |  |  | 0.857 | 0.072 |
| Elective (≥24 hours) | 315 (48.3) | 40 (47.5) |  |  |
| Emergent (<90 min) | 246 (37.7) | 34 (40.5) |  |  |
| Urgent (<24 hours) | 91 (14.0) | 10 (12.0) |  |  |
| Disease extent* |  |  | 0.814 | 0.084 |
| 1 vessel disease | 298 (45.7) | 41 (47.9) |  |  |
| 2 vessel disease | 226 (34.6) | 26 (30.7) |  |  |
| 3 vessel disease | 129 (19.7) | 18 (21.4) |  |  |
| LMCA | 46 (7.1) | 4 (4.4) | 0.482 | 0.113 |
| LAD | 506 (77.5) | 69 (81.0) | 0.490 | 0.086 |
| LCX | 247 (37.8) | 35 (41.9) | 0.508 | 0.084 |
| RCA | 308 (47.2) | 39 (46.0) | 0.847 | 0.025 |
| In stent restenosis | 17 (2.6) | 2 (2.7) | 0.939 | 0.008 |
| Chronic total occlusion | 45 (6.8) | 5 (5.8) | 0.702 | 0.044 |
| Bifurcation | 65 (9.9) | 10 (11.4) | 0.697 | 0.048 |
| Moderate to severe calcified lesions | 66 (10.1) | 8 (9.7) | 0.889 | 0.014 |
| Number of lesions stented | 1.6±0.9 | 1.7±0.9 | 0.781 | 0.033 |
| Total stent length (mm) | 43.1±28.1 | 43.3±25.5 | 0.962 | 0.005 |
| Average stent diameter (mm) | 3.2±0.4 | 3.1±0.4 | 0.135 | 0.184 |
| Complete revascularization | 517 (79.2) | 77 (91.0) | 0.050 | 0.335 |
| Contrast amount (mL) | 181.9±66 | 185.5±69.9 | 0.727 | 0.052 |
| Radial only | 432 (66.2) | 55 (64.6) | 0.807 | 0.032 |
| Serious complications** | 10 (1.5) | 0 (0.0) | 0.237 | 0.173 |
| Vascular complications† | 6 (0.9) | 0.0 (0.0) | 0.302 | 0.136 |
| Data are presented as the mean ± SD or N (%). | | | | |
| *A left main lesion is considered as a composite of LAD and LCX lesions. | | | | |
| ** Cardiogenic shock, transient hypotension, pulmonary edema, cardiac tamponade, acute limb ischemia, renal injury (including dialysis) and temporary mechanical ventilation | | | | |
| † Hematoma, AV fistula, pseudoaneurysm, deep vein thrombosis and infection | | | | |
| BMI, body mass index; CKD, chronic kidney disease; eGFR, estimated glomerular filtration rate; NSTEMI, non-ST segment elevation myocardial infarction; STEMI, ST segment elevation myocardial infarction; ARB, angiotensin receptor blocker; ACEI, angiotensin converting enzyme inhibitor; PPI, proton-pump inhibitor | | | | |

| Table S3. Baseline clinical characteristics of the patients with very complex coronary lesions | | | |
| --- | --- | --- | --- |
|  | Standard-dose | Low-dose |  |
|  | N=336 | N=57 | p-value |
| Clinical characteristics |  |  |  |
| Age (year) | 61.7±11.5 | 64±9.7 | <0.001 |
| Age ≥60 years | 183 (54.5) | 39 (68.4) | 0.069 |
| Male sex | 287 (85.4) | 45 (78.9) | 0.038 |
| BMI (kg/m^2^) | 25±3.4 | 24.2±2.8 | 0.304 |
| Smoking |  |  | 0.013 |
| Never | 129 (38.4) | 17 (29.8) |  |
| Former | 52 (15.5) | 18 (31.6) |  |
| Current | 155 (46.1) | 22 (38.6) |  |
| Comorbidities |  |  |  |
| Hypertension | 172 (51.2) | 33 (57.9) | 0.427 |
| Diabetes | 119 (35.4) | 16 (28.1) | 0.353 |
| Atrial fibrillation | 10 (3.0) | 2 (3.5) | 1.000 |
| CKD (eGFR ≤60 mL/min/1.73m^2^) | 45 (13.4) | 9 (15.8) | 0.781 |
| ESRD | 2 (0.6) | 1 (1.8) | 0.915 |
| Prior MI | 14 (4.2) | 1 (1.8) | 0.614 |
| Prior PCI | 27 (8) | 5 (8.8) | 1.000 |
| Prior PVD | 3 (0.9) | 0 (0.0) | 1.000 |
| HF | 7 (2.1) | 0 (0.0) | 0.577 |
| Clinical diagnosis |  |  | 0.513 |
| Stable angina | 14 (4.2) | 4 (7.0) |  |
| Unstable angina | 96 (28.6) | 12 (21.1) |  |
| NSTEMI | 95 (28.3) | 19 (33.3) |  |
| STEMI | 131 (39) | 22 (38.6) |  |
| Laboratory tests |  |  |  |
| eGFR (mL/min/1.73m^2^) | 82.2±21.3 | 82.8±23 | 0.840 |
| HbA1c (%) | 6.9±1.5 | 6.5±1.2 | 0.090 |
| Total cholesterol (mg/dL) | 182.8±51 | 180.3±42.1 | 0.727 |
| LDL cholesterol (mg/dL) | 113.9±44.5 | 102.1±33.7 | 0.057 |
| Triglyceride (mg/dL) | 164.2±120.2 | 134.7±66.6 | 0.072 |
| HDL cholesterol (mg/dL) | 44.4±10.4 | 45.8±9.9 | 0.358 |
| Medications |  |  |  |
| Statin | 325 (96.7) | 55 (96.5) | 1.000 |
| Beta blocker | 199 (59.2) | 42 (73.7) | 0.054 |
| ACEI/ARB | 191 (56.8) | 35 (61.4) | 0.618 |
| PPI | 164 (48.8) | 36 (63.2) | 0.063 |
| PARIS bleeding score | 4.3±2.2 | 4.5 ±1.7 | 0.526 |
| PARIS coronary thrombotic event score | 2.9±1.3 | 2.8±1.4 | 0.750 |
| DAPT score | 1.4±1.4 | 1.3±1.5 | 0.731 |
| Discontinuation of standard-dose ticagrelor-based DAPT | 208 (61.9) | 57 (100) | <0.001 |
| Duration for standard-dose ticagrelor-based DAPT | 406.5±150.2 | 155.2±98.2 | <0.001 |
| Discontinuation of ticagrelor-based DAPT* | 203 (60.4) | 45 (78.9) | 0.011 |
| Duration for ticagrelor-based DAPT* | 376 [354, 421] | 369 [324, 396] | 0.065 |
| Angiography and lesions |  |  |  |
| PCI situation |  |  | 0.382 |
| Elective (≥24 hours) | 165 (49.1) | 24 (42.1) |  |
| Emergent (<90 min) | 121 (36.0) | 26 (45.6) |  |
| Urgent (<24 hours) | 50 (14.9) | 7 (12.3) |  |
| Disease extent** |  |  | 0.096 |
| 1 vessel disease | 59 (17.6) | 8 (14.0) |  |
| 2 vessel disease | 143 (42.6) | 33 (57.9) |  |
| 3 vessel disease | 134 (39.9) | 16 (28.1) |  |
| LMCA | 47 (14.0) | 6 (10.5) | 0.619 |
| LAD | 279 (83.0) | 50 (87.7) | 0.489 |
| LCX | 193 (57.4) | 37 (64.9) | 0.361 |
| RCA | 208 (61.9) | 33 (57.9) | 0.669 |
| In stent restenosis | 8 (2.4) | 4 (7.0) | 0.143 |
| Chronic total occlusion | 45 (13.4) | 7 (12.3) | 0.986 |
| Bifurcation with side branch | 61 (18.2) | 13 (22.8) | 0.517 |
| Moderate to severe calcified lesion | 63 (18.8) | 17 (29.8) | 0.081 |
| Numbers of lesions | 1.8±0.9 | 2.5±1.1 | <0.001 |
| Total stent length (mm) | 54.9±31.9 | 64.9±29.6 | 0.027 |
| Average stent diameter (mm) | 3.1±0.4 | 3.0±0.4 | 0.074 |
| Complete revascularization | 252 (75.0) | 52 (91.2) | <0.001 |
| Procedure |  |  |  |
| Contrast amount (mL) | 198.5±73.1 | 187.7±53.4 | 0.289 |
| Radial access only | 213 (63.4) | 43 (75.4) | 0.106 |
| Serious complications† | 5 (1.5) | 0 (0.0) | 0.773 |
| Vascular complications†† | 4 (1.2) | 0 (0.0) | 0.909 |
| Data are presented as the mean ± SD or N (%). | | | |
| Data with a skewed distribution are presented as the median value [Interquartile range] | | | |
| * Discontinuation of any type of ticagrelor-based DAPT (either standard-dose or low-dose) and the duration of time for which any type of ticagrelor-based DAPT was used | | | |
| **A left main lesion was considered a composite of LAD and LCX lesions. | | | |
| † Cardiogenic shock, transient hypotension, pulmonary edema, cardiac tamponade, acute limb ischemia, renal injury (including dialysis) and temporary mechanical ventilation | | | |
| †† Hematoma, AV fistula, pseudoaneurysm, deep vein thrombosis and infection | | | |
| BMI, body mass index; CKD, chronic kidney disease; eGFR, estimated glomerular filtration rate; NSTEMI, non-ST segment elevation myocardial infarction; STEMI, ST segment elevation myocardial infarction; ARB, angiotensin receptor blocker; ACEI, angiotensin converting enzyme inhibitor; PPI, proton-pump inhibitor; PCI, percutaneous coronary intervention; LMCA, left main coronary artery disease; LAD, left anterior descending artery; LCX, left circumflex artery; RCA, right coronary artery; MACE, major adverse cardiovascular event; CV, cardiovascular | | | |

| Table S4. Clinical outcomes of patients with very complex coronary lesions | | | | | | |
| --- | --- | --- | --- | --- | --- | --- |
|  | Standard-dose | Low-dose | Univariate | | Multivariate* | |
|  | N=336 | N=57 | HR (95% CI) | p | HR (95% CI) | p |
| Entire follow-up | |  |  |  |  |  |
| MACE | 24 (7.1%) | 2 (3.5%) | 0.46 (0.11-1.96) | 0.295 | 0.68 (0.16-2.92) | 0.608 |
| Bleeding events | 1 (0.3%) | 2 (3.5%) | 12.0 (1.12-137) | 0.040 | - | - |
| NACE | 25 (7.4%) | 4 (7.0%) | 0.92 (0.32-2.63) | 0.872 | 1.69 (0.55-5.13) | 0.357 |
| Beyond 6 months | |  |  |  |  |  |
| MACE | 14 (4.2%) | 2 (3.5%) | 0.78 (0.18-3.41) | 0.736 | 1.22 (0.28-5.39) | 0.794 |
| Bleeding events | 1 (0.3%) | 2 (3.5%) | 12.0 (1.12-137) | 0.040 | - | - |
| NACE | 15 (4.5%) | 4 (7.0%) | 1.52 (0.50-4.58) | 0.458 | 1.79 (0.59-5.45) | 0.307 |
| *Multivariate model includes age, sex, BMI, current smoking, diabetes, hypertension, eGFR, LDL cholesterol, prior PCI, clinical diagnosis, atrial fibrillation, medications and lesion characteristics | | | | | | |
| The multivariate model was reduced using a backward variable selection procedure (criterion p>0.05). | | | | | | |
| MACE, major adverse cardiovascular event; NCE, net clinical event | | | | | | |

| Table S5. Numbers of clinical events and univariate and multivariate Cox proportional hazard models of the use of low-dose ticagrelor-based DAPT for the clinical events at 1 year after PCI | | | | | | | | | | |
| --- | --- | --- | --- | --- | --- | --- | --- | --- | --- | --- |
|  |  |  | Unweighted cohort | | | | Weighted cohort | | | |
|  | standard-dose | low-dose | Univariate | | Multivariate* | | Univariate | | Multivariate* | |
|  | N=657 | N=115 | HR (95% CI) | p | HR (95% CI) | p | HR (95% CI) | p | HR (95% CI) | p |
| MACE | 32 (4.9%) | 2 (1.7%) | 0.34 (0.08-1.42) | 0.140 | 0.43 (0.10-1.82) | 0.254 | 0.28 (0.04-1.86) | 0.186 | 0.38 (0.06-2.61) | 0.326 |
| Death | 15 (2.3%) | 0 (0.0%) | - | - | - | - | - | - | - | - |
| CV death | 5 (0.8%) | 0 (0.0%) | - | - | - | - | - | - | - | - |
| Myocardial infarction | 3 (0.5%) | 1 (0.9%) | - | - | - | - | - | - | - | - |
| Ischemic stroke | 1 (0.2%) | 1 (0.9%) | - | - | - | - | - | - | - | - |
| Repeat revascularization | 15 (2.3%) | 1 (0.9%) | 0.36 (0.05-2.73) | 0.323 | 0.52 (0.07-3.97) | 0.689 | 0.11 (0.02-8.17) | 0.317 | 0.11 (0.01-8.17) | 0.314 |
| Stent thrombosis | 1 (0.2%) | 0 (0.0%) | - | - | - | - | - | - | - | - |
| Bleeding events | 6 (0.9%) | 2 (1.7%) | 1.85 (0.37-9.14) | 0.453 | 2.01 (0.40-9.93) | 0.394 | 2.67 (0.53-13.4) | 0.231 | 3.01 (0.60-15.0) | 0.180 |
| NACE | 37 (5.6%) | 4 (3.5%) | 0.59 (0.21-1.67) | 0.322 | 0.71 (0.25-2.02) | 0.523 | 0.68 (0.21-2.18) | 0.517 | 0.78 (0.24-2.51) | 0.673 |
| *Multivariate model includes age, sex, BMI, current smoking, diabetes, hypertension, eGFR, LDL cholesterol, prior PCI, clinical diagnosis, atrial fibrillation, medications and lesion characteristics | | | | | | | | | | |
| The multivariate model was reduced using a backward variable selection procedure (criterion p>0.05). | | | | | | | | | | |
| MACE, major adverse cardiovascular event; CV cardiovascular; NACE, net adverse clinical event; DAPT, dual antiplatelet agent therapy | | | | | | | | | | |
